# Hyperaldosteronism Presenting With Unilateral Blindness: A Case Report

**DOI:** 10.7759/cureus.43195

**Published:** 2023-08-09

**Authors:** Sebastian L Manuel, Mark Kender

**Affiliations:** 1 Internal Medicine, Saint Luke's Hospital, Easton, USA; 2 Internal Medicine, Saint Luke’s Hospital, Easton, USA; 3 Medicine, Temple School of Medicine, Bethlehem, USA

**Keywords:** blindness unilateral, resistant hypertension, primary hyperaldosteronism, hyperaldosteronism, blind, central retinal vein occlusion (crvo)

## Abstract

Hyperaldosteronism is a common cause of secondary hypertension. It has been classically associated with the clinical triad of hypertension, unexplained hypokalemia, and metabolic alkalosis. We present a case of a 66-year-old man who experienced blindness, hypokalemia, and hypertension that was resistant to anti-hypertension medications. He was found to have a retinal detachment and central retinal vein occlusion (CRVO). Laboratory evaluation revealed a marked elevation of plasma aldosterone activity and suppressed renin. A computerized tomography (CT) abdomen was subsequently ordered, which revealed bilateral adrenal nodules. Adrenal vein sampling was performed, which confirmed bilateral hyperfunctioning adrenal nodules. He was successfully treated with spironolactone. CRVO in the setting of hyperaldosteronism is an uncommon presentation.

## Introduction

Primary aldosteronism (PA) was found to be a common cause of secondary hypertension [1,2]. A meta-analysis estimated the prevalence to vary from 3.2% to 12.7% in the primary care setting [2]. It has been classically associated with the clinical triad of hypertension, unexplained hypokalemia, and metabolic alkalosis [3]. This case report elucidates a unique presentation of primary aldosteronism. It is about a 66-year-old male who initially presented with blindness and was found to have uncontrolled hypertension despite being on three hypertension medications. He was found to have a central retinal vein occlusion (CRVO) and a posterior vitreous detachment. He was eventually found to have bilateral hyperfunctioning adrenal glands. Not much literature has been published on this presentation. One similar case report was found about a 44-old-year old male presenting with blindness of the left eye, which was found to be secondary to CRVO from an aldosterone-secreting adrenal adenoma [4]. This case report provides a review of the approach to primary aldosteronism and explores the current data about the association between elevated aldosterone levels and CRVO. Written informed consent was obtained from the patient for the publication of this case report.

## Case presentation

**Figure 1:**
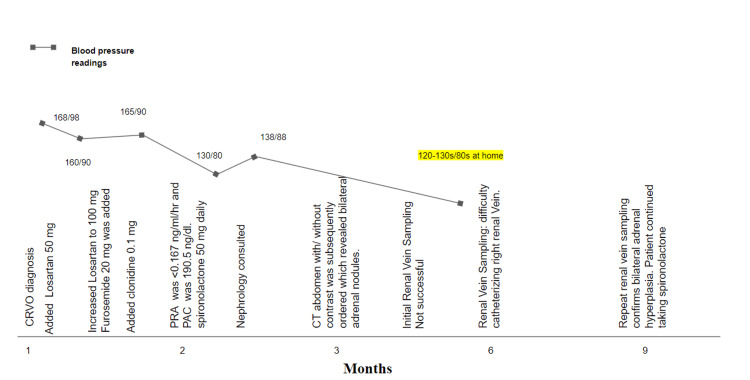
Timeline of events PRA, plasma renin activity; PAC, plasma aldosterone concentration.

This is a case of a 66-year-old male with hypertension and hyperlipidemia who presented at the internal medicine clinic. The timeline of clinical events is illustrated in Figure 1. He was recently diagnosed with central retinal vein occlusion (CRVO) accompanied by macular edema, leading to functional blindness in his right eye. During this period, he reported increased floaters in the right eye for two weeks. Subsequently, he experienced severe blurring of vision in the right eye. He was evaluated by ophthalmology and had a recent follow-up, revealing a visual acuity of 20/800 in the right eye and 20/20 in the left eye. According to ophthalmology notes, the patient's right eye exhibited features such as posterior vitreous detachment, CRVO, scattered dot hemorrhages, and cotton wool spots in the macular region. Additionally, the patient demonstrated scattered retinal hemorrhages across all quadrants. In contrast, his left eye displayed normal findings without any abnormalities. Aflibercept injections failed to improve his clinical condition. A review of his symptoms revealed no significant findings. Notably, he has no history of deep vein thrombosis, pulmonary embolism, or any indications of a hypercoagulable state. Furthermore, there is no record of prior medical issues such as glaucoma or cataract. He is currently prescribed labetalol 300 mg twice daily. The patient maintains a generally healthy lifestyle.

The patient was generally healthy. He maintained a low salt intake, was a nonsmoker, and only consumed alcohol occasionally. He was obese with a BMI of 31; however, he was trying to lose weight with calorie restriction and exercise. His blood pressure at this time was 168/98. Initially, the patient was managed by adding losartan 50 mg once daily. He was advised to continue lifestyle modification and restrict salt to 2 g daily. The patient was instructed to obtain blood pressure readings at home and follow up after 1 week. 

On follow-up two weeks later, he continued to have systolic BP in the 150-160 mmHg range. He was compliant with all his medications. Losartan was increased to 100 mg daily, and furosemide 20 mg once a day was started. At this point, he was on labetalol, losartan, and furosemide. Workup for secondary causes of hypertension was initiated by getting morning cortisol, complete blood count, complete metabolic panel, thyroid stimulating hormone, plasma aldosterone concentration (PAC), and plasma renin activity (PRA). The thrombosis panel was negative. Thyroid levels were negative. The patient was found to have low potassium at 3.4 mmol/l. EGFR was stable at 80 ml/min/1.73 sqm. AM cortisol was found to be normal. The anion gap was normal at 4 mmol/L, and bicarbonate was slightly elevated at 27 mmol/L. 

The patient returned for a follow-up appointment after one week. His blood pressure was noted to be persistently elevated at 165/100 despite being on three antihypertensive agents. Clonidine 0.1 mg twice daily was also added.

In the interim, the PRA was found to be <0.167 ng/ml/hr, and PAC was 190.5 ng/dl. This confirmed the suspicion of primary aldosteronism. Furosemide was replaced with spironolactone 50 mg daily. Two weeks after starting this medication, his systolic blood pressure improved to the 130s range during subsequent visits.

**Figure 2:**
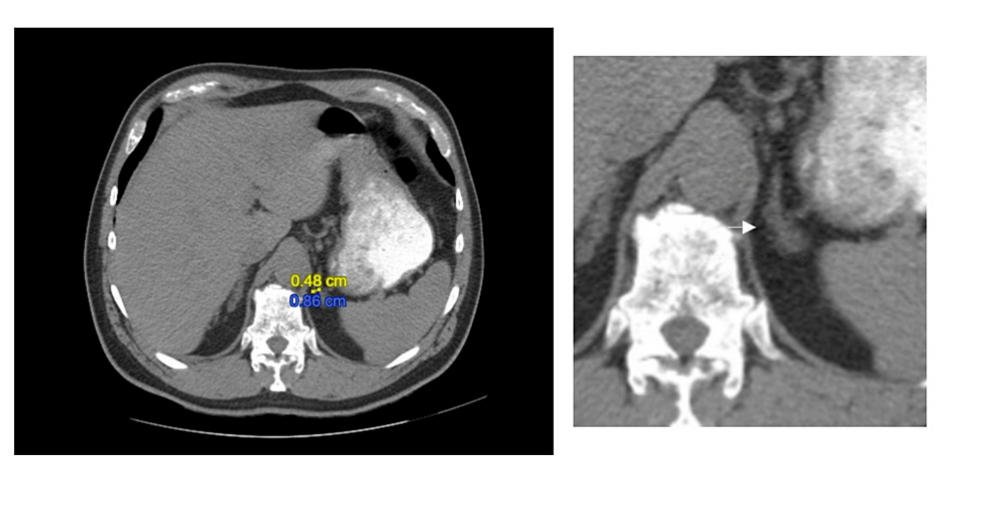
Left adrenal nodule measuring 9 mm x 5 mm. The panel on the right shows a zoomed-in image.

**Figure 3:**
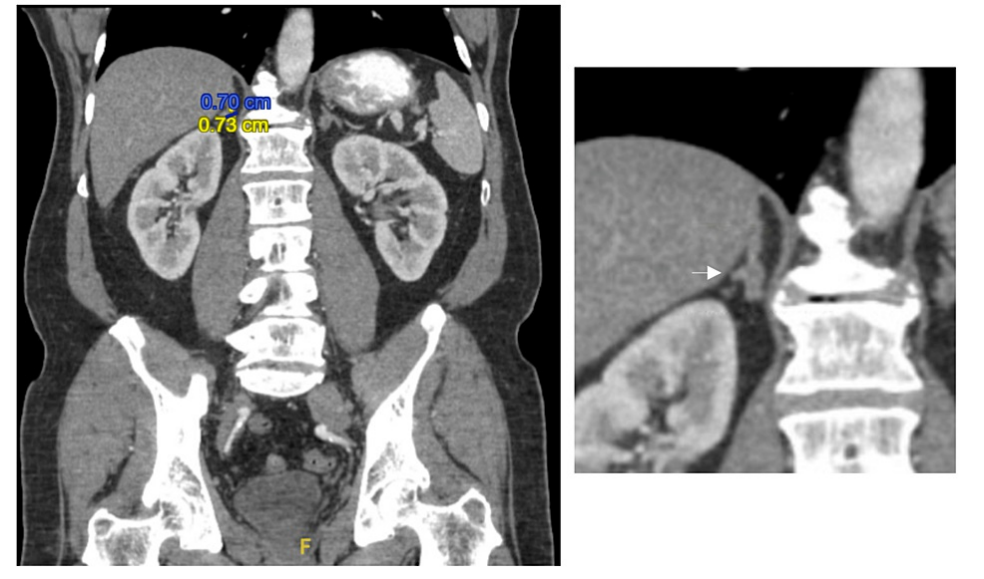
Right adrenal nodule measuring 7 mm x 7 mm. The panel on the right shows a zoomed-in image.

Nephrology was consulted at this point. A CT abdomen without contrast was subsequently ordered, revealing bilateral adrenal nodules. A left adrenal nodule measuring 9 mm x 5 mm and a right adrenal nodule measuring 7 mm x 7 mm were identified (Figures 2, 3). The patient agreed to undergo renal vein sampling to determine laterality. Spironolactone was discontinued six weeks prior to renal vein sampling, as it has the potential to alter the test results.

 

Bilateral renal vein sampling was done six weeks later (Figure 1). Aldosterone was markedly elevated on the left side after cosyntropin stimulation, which reached as high as 2133.6 ng/dl. Samples from the right renal vein did not show aldosterone elevation; however, due to difficulty with catheterization on that side, the accuracy of the data was questioned. Because of this, repeat renal vein sampling was done in June; however, that also was unsuccessful due to the difficulty of catheterizing the right renal vein. Spironolactone was then restarted at this time. 

The patient was then referred to the interventional radiology group at a university hospital where a successful renal vein sampling was done. No laterality was found with an index of 2.6. The patient was deemed to have bilateral hyperfunctioning adrenal glands. Hence, the patient was placed on spironolactone 50 mg once daily. Today his blood pressures are well controlled. The last few office visits showed that blood pressure has been well controlled at 120/80 mmHg. Unfortunately, he was not able to regain vision in his right eye. 

## Discussion

Primary aldosteronism has been associated with the clinical triad of hypertension, unexplained hypokalemia, and metabolic alkalosis [3]. Our patient presented with uncontrolled hypertension despite being on labetalol 300 mg twice daily, furosemide 20 mg once a day, losartan 100 mg once a day, and clonidine 0.1 mg twice a day. He also presented with hypokalemia with a potassium level of 3.4 mmol/l. The patient had mild bicarbonate elevation at 27. It is estimated that only 9 to 37% of patients with primary aldosteronism present with hypokalemia [1]. 

This patient also presented with CRVO, which caused blindness in his right eye. In our review of the literature, only one other case of hyperaldosteronism presenting with CRVO was identified [4]. Aldosterone has been found to be a mediator of severity in retinal vascular disease. In studies done on rats, systemic administration of aldosterone had a negative correlation with retinal ganglion cell survival [5]. The mechanism by which aldosterone increases the severity of retinal vascular disease includes microvascular cellular dysfunction, increased inflammation, and Müller glial dysfunction [6]. The patient was found to have a very high plasma aldosterone concentration of 190.5 ng/dl. 

According to the 2016 clinical practice guidelines from the Endocrine Society, plasma aldosterone concentration (PAC) and plasma renin activity (PRA) or plasma renin concentration (PRC) are used to screen for hyperaldosteronism [7]. PAC greater than or equal to 10 ng/dl and PRC or PRA less than the lower limit would be indicative of primary aldosteronism. A PAC greater than 20 and spontaneous hypokalemia would also be enough to make a formal diagnosis of primary aldosteronism. For the patient in the case report, PRA was <0.167 ng/ml/hr and PAC was 190.5 ng/dl. For comparison, normal PRA is 0.7- 3.3 ng/ml/hr, and normal PAC is 7-30 ng/dl. He also presented with spontaneous hypokalemia with a potassium level of 3.4 mmol/l. He met the criteria for primary aldosteronism. 

Adrenal CT scan without contrast is the imaging of choice and has been noted to have a superior spatial resolution compared to magnetic resonance imaging (MRI) [8]. Adrenal vein sampling is typically done when to differentiate between unilateral and bilateral disease, particularly when both adrenal glands have abnormal findings. To confirm successful catheterization, the adrenal vein to IVC ratio should be at least 5:1 [9]. For our patient, in the first two attempts, the IVC ratio was less than 5:1 for the right side. Unilateral diseases are usually associated with fourfold greater aldosterone levels on the side of the tumor [9,10]. Our patient had a laterality index of 2, which points more toward bilateral hyperplasia.

Our patient had multiple attempts at catheterizing the right renal vein. He was referred to the interventional radiology team at a university hospital where it was finally done successfully. Although left adrenal vein catheterization has a near 100% success rate, the right adrenal vein has been shown to be more difficult due to some variability in anatomy [11]. 

## Conclusions

This case report highlights the importance of diagnosing the underlying cause of hypertension. We were able to describe a unique presentation of primary hyperaldosteronism, which initially presented as unilateral blindness. The classic findings of hypokalemia and resistant hypertension were also present. The patient's markedly elevated plasma aldosterone concentration could have also contributed to a worse outcome on the patient's vision since some studies suggest a possible link between high aldosterone blood concentrations and worse retinal ganglion cell survival. 
